# Comparison of IgA, IgG, and Neutralizing Antibody Responses Following Immunization With Moderna, BioNTech, AstraZeneca, Sputnik-V, Johnson and Johnson, and Sinopharm’s COVID-19 Vaccines

**DOI:** 10.3389/fimmu.2022.917905

**Published:** 2022-06-21

**Authors:** Tomabu Adjobimey, Julia Meyer, Leander Sollberg, Michael Bawolt, Christina Berens, Peđa Kovačević, Anika Trudić, Marijo Parcina, Achim Hoerauf

**Affiliations:** ^1^Institute of Medical Microbiology, Immunology and Parasitology (IMMIP), University Hospital Bonn, Bonn, Germany; ^2^Faculté des Sciences et Techniques (FAST), Université d’Abomey Calavi, Abomey-Calavi, Bénin; ^3^Medical Intensive Care Unit, University Clinical Center of Republic of Srpska, Banja Luka, Bosnia and Herzegovina; ^4^Faculty of Medicine, University of Novi Sad, Novi Sad, Serbia; ^5^Institute for Pulmonary Diseases of Vojvodina, Sremska Kamenica, Serbia; ^6^Bonn-Cologne Site, German Center for Infectious Disease Research (DZIF), Bonn, Germany

**Keywords:** SARS-CoV-2, COVID-19 vaccines, neutralizing antibodies, ELISA, IgA, IgG

## Abstract

In an ongoing multinational trial, we obtained blood samples from 365 volunteers vaccinated with mRNA vaccines (Moderna, BioNTech), viral DNA-vectored vaccines (AstraZeneca, Sputnik-V, and Johnson and Johnson), or the attenuated virus vaccine from Sinopharm. After collecting reactogenicity data, the expression of S-Protein binding IgG and IgA was analyzed using an automated sandwich ELISA system. Serum neutralizing potentials were then investigated using an ACE-2-RBD neutralizing assay. Moderna’s vaccine induced the highest amounts of SARS-CoV-2 specific neutralizing antibodies compared to the other groups. In contrast, Sinopharm and Johnson and Johnson’s vaccinees presented the lowest SARS-CoV-2-specific antibody titers. Interestingly, moderate to high negative correlations between age and virus-specific IgG expression were observed in the Johnson and Johnson (ρ =-0.3936) and Sinopharm (ρ =-0.6977) groups according to Spearman’s rank correlation analysis. A negative correlation was seen between age and IgA expression in the Sputnik-V group (ρ =-0.3917). The analysis of virus neutralization potentials in age categories demonstrated that no significant neutralization potential was observed in older vaccinees (61and 80 years old) in the Sputnik-V Johnson and Johnson and Sinopharm vaccinees’ groups. In contrast, neutralization potentials in sera of Moderna, BioNTech, and AstraZeneca vaccinees were statistically comparable in all age categories. Furthermore, while the AstraZeneca vaccine alone induced moderate IgG and IgA expression, the combination with Moderna or BioNTech mRNA vaccines induced significantly higher antibody levels than a double dose of AstraZeneca and similar IgG expression and neutralization potential compared to Moderna or BioNTech vaccines used alone. These results suggest that mRNA vaccines are the most immunogenic after two doses. DNA vectored vaccines from AstraZeneca and Sputnik-V presented lower but significant antibody expression and virus neutralizing properties after two doses. The lowest antibody and neutralization potential were observed in the Sinopharm or Johnson and Johnson vaccinees. Especially elderly over 60 presented no significant increase in neutralizing antibodies after vaccination. The data also indicate that heterologous vaccination strategies combining the AstraZeneca DNA vectored vaccines and mRNA vaccines are more effective in the induction of neutralizing antibodies compared to their homologous counterparts.

## Introduction

Since it began in November 2019, the COVID-19 pandemic has caused significant morbidity and mortality worldwide and major social, educational, and economic disruptions. It is the most serious global health crisis ever experienced in modern history (1). Seniors and persons with comorbidities are at the highest risk for COVID-19 complications (2). Globally, as of April 8^th,^ 2022, there have been 494 587 638 confirmed cases of COVID-19, including 6 170 283 deaths, reported to the WHO (3). Recent data indicate increasing SARS-CoV-2 infection rates and COVID-19 in younger adults due to the occurrence of new virus variants (4, 5). In this context, vaccine rollout represents a tool of choice to fight the pandemic. Several groups developed vaccines to prevent the infection and control the pandemic. The amount of resources and the extraordinary speed of vaccine development against COVID-19 are unique in human history (6). While the WHO declared COVID-19 a pandemic in March 2020, less than nine months later, more than 60 vaccines entered clinical trials, with 13 in Phase III clinical trials at the end of 2020 (7). New technologies, accumulated expertise during the development of vaccines against related viruses like MERS-CoV or SARS-CoV-1, as well as existing production platforms have made this fulminant acceleration possible (8). As of April 5^th,^ 2022, a total of 11 250 782 214 vaccine doses have been administered worldwide (3). Currently available vaccines against COVID-19 include Messenger ribonucleic acid (mRNA) vaccines like Moderna’s Spikevax and Pfizer-BioNTech’s Comirnaty, recombinant adenovirus vectored vaccines such as Vaxzevria from Oxford-AstraZeneca, Janssen from Johnson & Johnson Pharm, and Sputnik-V from the Gamaleya Research Center in Russia. The Chinese pharmaceutical firm Sinopharm opted for a more classical approach using inactivated viruses and proposed the BIBP COVID-19-vaccine, also known as BBIBP-CorV. All these vaccine candidates have been authorized for human use in many countries (9). The approval of these vaccines offers a highly effective tool for the global control of the COVID-19 pandemic. While the high-speed vaccine development is undoubtedly a scientific and technological success, it has also raised concerns about safety and efficacy in the global population (10). In this context, independent evaluations of the safety and effectiveness of these vaccines are highly needed.

Humoral immune responses and particularly neutralizing antibodies are key elements of the adaptive immunity against acutely cytopathic viruses such as the SARS-CoV-2 (11–13). Gamma (IgG) and alpha (IgA) immunoglobulins are the first and second most abundant immunoglobins in human serum, respectively (14). There is increasing evidence that neutralizing responses correlate with protection against COVID-19 (15). Both IgG and IgA were reported to mediate viral neutralization in COVID-19 patients, and their neutralization potential is the key mechanism supporting the efficacy of convalescent plasma in the treatment of severe COVID-19 patients (16–19). While IgG is the main antibody in the blood and most tissues, IgA is the most abundant antibody on mucosal surfaces (14, 20), including the respiratory mucosa, main entry, and replication site of SARS-CoV-2 in the human body (20–23). Investigations in influenza and SARS-CoV-2 infections suggested higher antiviral properties for IgA in comparison to IgG (24, 25), suggesting a key role for IgA in protective immunity against SARS-CoV-2. However, limited data exist on IgG and IgA responses after COVID-19 vaccinations. In the present study, we compared SARS-CoV-2 spike antigen-specific serum IgA and IgG expression as well as virus neutralization potential in individuals vaccinated with five different COVID-19 vaccines, including mRNA vaccines from Pfizer-BioNTech and Moderna, viral DNA vectored vaccines from Johnson & Johnson, Oxford-AstraZeneca, and Sputnik-V, as well as the inactivated virus vaccine from Sinopharm (BIBP COVID-19-vaccine). In the present study, we investigated the humoral immune response in blood samples from volunteers vaccinated with mRNA vaccines (Moderna, BioNTech), viral DNA-vectored vaccines (AstraZeneca, Sputnik-V, and Johnson and Johnson), or the inactivated virus vaccine from Sinopharm.

## Material and Methods

### Participants, Sample Collection, and Ethics

The study was conducted between January 2021 and October 2021 at the Institute of Medical Microbiology, Immunology and Parasitology (IMMIP) of the University Hospital of Bonn and is part of an ongoing survey. Volunteers were recruited in Bonn (Germany), in Sremska Kamenica (Serbia), and in Banja Luka (Bosnia and Herzegovina). A total of 365 (122 men and 243 women) were included in the study. Each volunteer recruited for the study gave informed consent to participate. Ethics approval for the study was obtained from the ethical boards of the University Hospital Bonn (Lfd.Nr.439/20) and the Faculty of Medicine of the University of Novi Sad (FN.198/02). Epidemiological and clinical characteristics of study participants are summarized in **Table 1**. Venous blood was collected 14-30 days after the last vaccination dose using the S-Monovette SERUM GEL blood collection system (Sarstedt AG, Nümbrecht, Germany). Characteristics of all vaccines investigated during the study are listed in **Table 2**. Donors with immune deficiency or using immunosuppressive treatment were excluded from the analyses.

**Table 1:** Epidemiological and clinical characteristics of study participants.

	Moderna	BioNTech/Pfizer	AstraZeneca	Johnson and Johnson	Sputnik-V	Sinopharm	AstraZeneca+ Moderna	AstraZeneca+ BioNtech	Total vaccinated	Controls
**Sample size (N=)**	41	92	52	34	35	28	43	40	365	30
**Mean age** **(Min-Max, Mean±SD) in years**	18-85 (42.1 ± 15.8)	20-88 (44.3 ± 18.5)	20-82 (42.9. ± 16.9)	20-71 (35.5 ± 15.6)	18-78 (36.0 ± 10.7)	26-74 (33.6 ± 15.0)	20-6 (42.1. ± 20.2)	20-98 (45.9. ± 25.2)	18-98 (45.7. ± 17.7)	18-78 (33.9 ± 25.3)
**Sex (M/F)**	13/28	37/55	14/38	23/11	11/24	11/17	6/37	7/33		12/18
**Hypertension (N=)**	5	25	8	5	3	4	2	6	58	3
**BMI (Mean±SD)**	24.4±4.6	26.7±7.5	26.4±5.0	25.2 ±6.2	25.6±4.5	26.5±4.5	22.7±6.2	25.6±5.7	25.3±5.9	25.4± 3.5

**Table 2:** Characteristics of investigated vaccines.

Vaccine Name	Manufacturers	Active component First dose	Active component Second dose	Type	References
**mRNA-1273**	Moderna, Massachusetts, USA	100 μg mRNA	100 μg mRNA	mRNA-based	Baden LR, El Sahly HM, Essink B, Kotloff K, Frey S, Novak R, et al. Efficacy and Safety of the Mrna-1273 Sars-Cov-2 Vaccine. *N Engl J Med* (2021) 384(5):403-16. Epub 20201230. doi: 10.1056/NEJMoa2035389.
**Comirnaty**	BioNTech SE, Mainz Germany Pfizer Inc, New York, USA	30 µg mRNA	30 µg mRNA	mRNA-based	Polack FP, Thomas SJ, Kitchin N, Absalon J, Gurtman A, Lockhart S, et al. Safety and Efficacy of the Bnt162b2 Mrna Covid-19 Vaccine. *N Engl J Med* (2020) 383(27):2603-15. Epub 20201210. doi: 10.1056/NEJMoa2034577.
**Vaxzevria**	AstraZeneca Corporation, Cambridge, UK	5×10^10^ viral particles	5×10^10^ viral particles	Adenovirus-vectored	Falsey AR, Sobieszczyk ME, Hirsch I, Sproule S, Robb ML, Corey L, et al. Phase 3 Safety and Efficacy of Azd1222 (Chadox1 Ncov-19) Covid-19 Vaccine. *N Engl J Med* (2021) 385(25):2348-60. Epub 20210929. doi: 10.1056/NEJMoa2105290.
**Ad26.COV2.S**	Johnson & Johnson, New Jersey, USA	5×10^10^ viral particles	–	Adenovirus-vectored	Sadoff J, Gray G, Vandebosch A, Cardenas V, Shukarev G, Grinsztejn B, et al. Safety and Efficacy of Single-Dose Ad26.Cov2.S Vaccine against Covid-19. *N Engl J Med* (2021) 384(23):2187-201. Epub 20210421. doi: 10.1056/NEJMoa2101544.
**Gam-COVID-Vac**	Gamaleya National Research Centre for Epidemiology and Microbiology, Moscow, Russia** **	(1.0±0.5) x 10^11^ viral particles	(1.0±0.5) x 10^11^ viral particles	Adenovirus-vectored	Logunov DY, Dolzhikova IV, Shcheblyakov DV, Tukhvatulin AI, Zubkova OV, Dzharullaeva AS, et al. Safety and Efficacy of an Rad26 and Rad5 Vector-Based Heterologous Prime-Boost Covid-19 Vaccine: An Interim Analysis of a Randomised Controlled Phase 3 Trial in Russia. *Lancet* (2021) 397(10275):671-81. Epub 20210202. doi: 10.1016/S0140-6736(21)00234-8.
**BBIBP-CorV**	Sinopharm, Beijing, China	6.5 U (4 μg) of inactivated SARS-CoV-2 antigens	6.5 U (4 μg) of inactivated SARS-CoV-2 antigens	Inactivated virus	WHO. Background Document on the Inactivated Covid-19 Vaccine Bibp Developed by China National Biotec Group (Cnbg), Sinopharm, 7 May 2021. (2021).

### Reactogenicity Analysis

Reactogenicity investigations were done using questionnaires designed by the investigators and delivered to the participants. The survey was done 2 to 3 weeks after each vaccine shot. Participants who agreed to participate gave written consent and filled a form with their demographics, including sex, age, earlier infection with SARS-CoV-2, and the observed side effects. All responses were included anonymously.

### Enzyme-Linked Immunosorbent Assay (ELISA) for the Detection of SARS-COV-2 Specific IgG and IgA

To detect the levels of SARS-CoV-2 specific antibodies, the Euroimmun SARS-CoV-2 IgG/IgA ELISA kit (Euroimmun, Lübeck, Germany) was used, according to the manufacturer’s instructions. Serum samples were diluted 1:101 in the provided sample buffer and incubated at 37° C for 60 min in a 96-well microtiter plate. Washings and incubation cycles were performed automatically using the predesigned program of Euroimmun’s Analyzer I automate. Optical densities (OD) were measured at 450 nm. SARS-CoV-2 specific immunoglobulin G and A expressions were calculated, and results were interpreted as per the manufacturer’s protocol.

### Neutralizing Antibody Level

To test the neutralizing potential of SARS-CoV-2 specific antibodies in the sera of vaccinated individuals, SARS-CoV-2 antibody neutralizing immunoassay kits (ThermoFisher Scientific) were used according to the manufacturer’s instructions. Briefly, 100 µL of controls and 1:5 diluted sera from fully vaccinated or unvaccinated individuals were added to the wells of microplates pre-coated with SARS-CoV-2 receptor-binding domain (RBD) protein. The plates were then incubated for 1 hour to allow neutralizing antibodies present in the samples to bind to RBD specifically. After 3 washes with the provided wash solution, 100 µL of biotinylated 1x ACE2 and samples were incubated for an additional 1h. After incubation, samples were washed 3 times, and 100 µL of 1x Streptavidin-HRP was added to each well. Plates were then incubated for an additional 30 min, and 50µL of stop solution containing 2N H2SO4 was added to stop the reaction. Signal development is indirectly proportional to the amount of specific neutralizing antibodies present. Plates were measured at 450 nm with the SpectraMax Pro plate reader (Molecular Devices), and neutralization potential was calculated according to the provided controls.

### Statistics

Descriptive demographic and clinical data analyses are presented as mean ± SD when continuous and as proportions (%) when categorical. All graphs were generated using GraphPad Prism 8 (La Jolla, CA, USA). p values were calculated using the Kruskal Wallis test. Dunnett’s multiple comparison test was used to compare all settings to the control (Unvaccinated), and Dunn’s *post hoc* test was used to compare all groups. Significance is accepted if p <0.05.

## Results

### Lowest Systemic Reactogenicity After Vaccination With Sinopharm’s BIBP COVID-19 Vaccine

**Figure 1:**
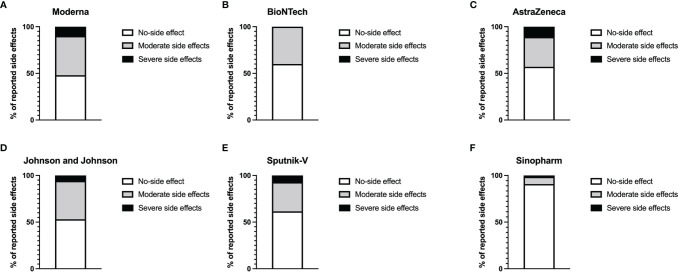
Level of reported systemic adverse reactions. Graphs represent the proportion of individuals presenting side effects in Moderna **(A)**, BioNTech **(B)**, AstraZeneca **(C)**, Johnson and Johnson **(D)**, Sputnik-V **(E)**, and Sinopharm **(F)** vaccinees. The white parts of the histograms indicate the percentage of persons with no systemic side effects. The grey portions represent the percentage of people with mild side effects, while the dark parts indicate the percentages of individuals with severe side effects.

We first analyzed the reactogenicity of the different vaccines using an appropriate questionnaire. The most common local symptoms were pain at the site of injection and skin rash. Minor systemic side effects were seen in all groups. More severe systemic effects, including musculoskeletal symptoms, fever, and headache for more than 3 days, were observed in the Moderna (10%), AstraZeneca (11%), Johnson and Johnson (5.9%), and Sputnik-V (7.2%) groups. No severe systemic effect was reported in the BioNTech group. Sinopharm’s BIBP COVID-19 vaccinees presented the lowest percentage of adverse reactions. Indeed, 93.2% % of the participants who received this vaccine declared having experienced no systemic side effects (**Figure 1**).

### Higher Expression of SARS-CoV-2-Specific IgG and IgA in mRNA Vaccinated Individuals

**Figure 2:**
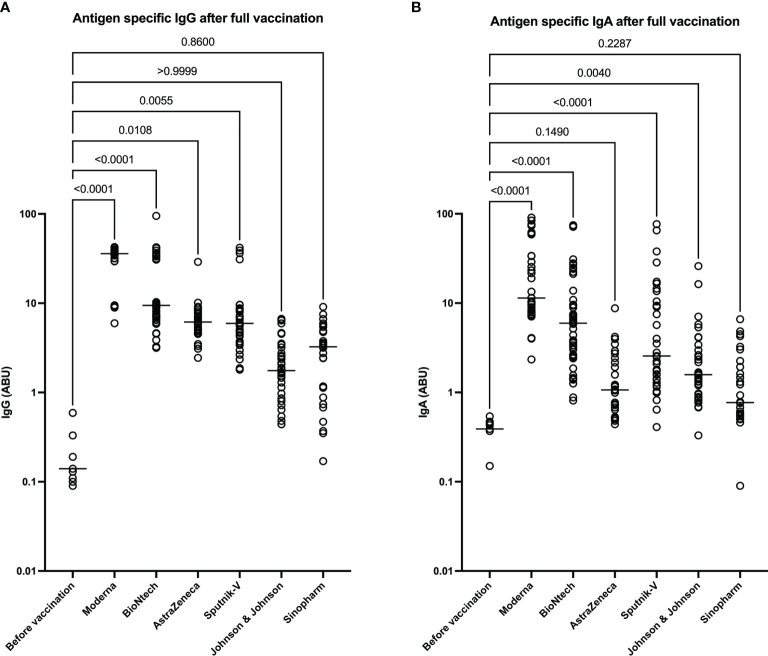
Higher expression of SARS-CoV-2 reactive IgG and IgA after immunization mRNA vaccines. Graphs represent the expression SARS-CoV-2 spike-protein binding IgG **(A)** and IgA **(B)** in arbitrary binding units (ABU). Each symbol represents individual donors. Indicated p values were calculated using the Kruskal-Wallis test followed by Dunnett’s *post hoc* to compare each group to the controls before vaccination. Bars represent the median antibody expression in each group. Significance is accepted if p <0.05.

To investigate the levels of SARS-CoV-2 specific antibody expression in the different groups of vaccinees, we analyzed S-protein specific IgG and IgA in the sera of vaccinated individuals 3-6 weeks after full vaccination. The results indicate that mRNA vaccines generally induced the highest amounts of SARS-CoV-2-reactive IgG and IgA. The Moderna vaccine induced slightly more IgG and IgA compared to the BioNTech vaccine. AstraZeneca and Sputnik-V induced comparable amounts of SARS-CoV-2 specific IgG. However, IgA expression was higher in the Sputnik-V group compared to the AstraZeneca group. The IgG expression in these two groups was significantly higher compared to the unvaccinated controls but lower compared to both mRNA vaccines. In contrast, the amount of SARS-CoV-2-specific IgG in the Johnson and Johnson and Sinopharm groups was moderately increased compared to the unvaccinated control group. However, differences to the control group were statistically not significant (p> 0.999 and p=0.860, respectively). However, while SARS-CoV-2 specific IgA was significant in the Johnson & Johnson group (p=0.004), no statistical difference was seen between Sinopharm and the control groups (p= 0.2287) in regard to SARS-CoV-2-specific IgA expression (**Figure 2**).

### Low Level of SARS-CoV-2-Specific Neutralizing Antibodies in Sera of Sinopharm and Johnson and Johnson Vaccinated Individuals

**Figure 3:**
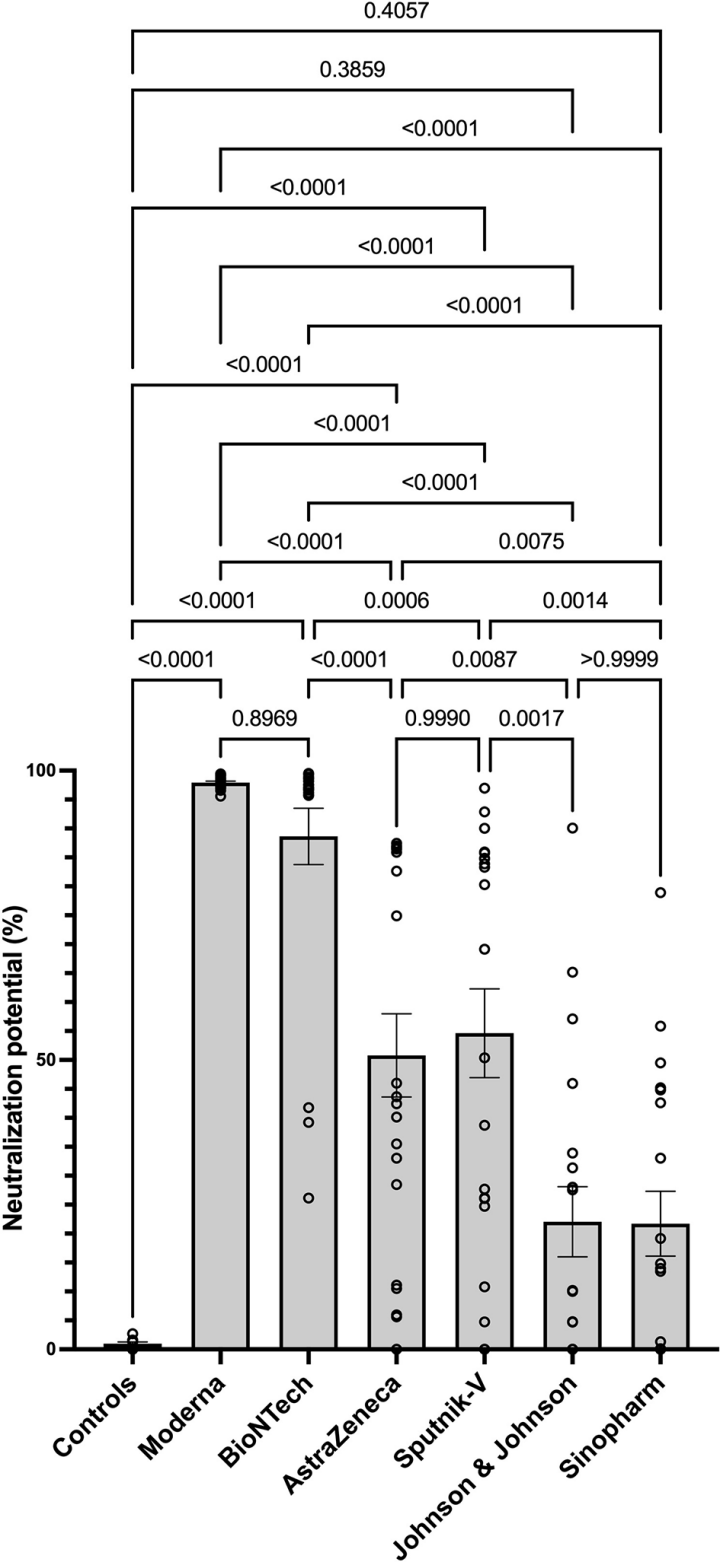
Higher neutralizing potential of mRNA vaccines in mRNA-vaccinated individuals. The graph represents the relative neutralizing antibody expression observed using sera from each vaccination group. Each symbol represents individual donors. Indicated p values were calculated using Kruskal-Wallis ‘test followed by Dunn’s comparison *post hoc* to compare all groups. Bars represent the mean ± SEM percentage of relative neutralizing antibody expression in each group. Significance is accepted if p <0.05.

Next, we determined the level of SARS-CoV-2-specific neutralizing antibodies in sera of vaccinated individuals. The data suggest Moderna and BioNTech groups exhibited the highest neutralization potential compared to the unvaccinated controls and all other groups. These data confirmed that mRNA COVID-19 vaccines induce antibodies with the highest neutralizing potential. In line with their antibody levels, AstraZeneca and Sputnik-V groups exhibited similar and significant neutralization potential compared to the unvaccinated controls. In contrast, no significant difference was seen between the Johnson and Johnson and Sinopharm groups and the control group in terms of SARS-CoV-2 neutralization potential (**Figure 3**).

### Negative Correlation Between Age and IgG Production in Johnson and Johnson and Sinopharm Vaccinees

**Figure 4:**
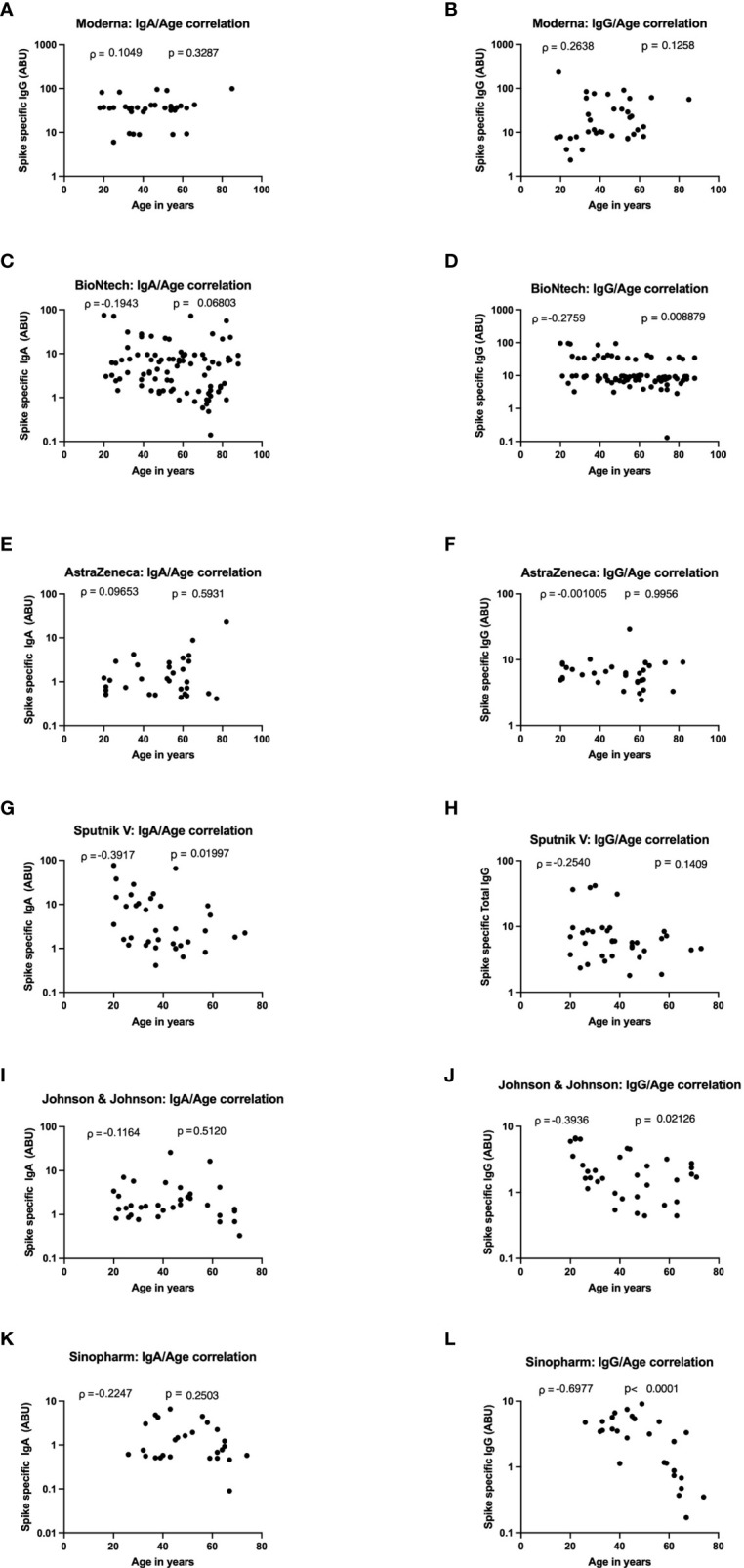
Strong age-dependent decrease of vaccine-induced antibody decrease in Sputnik-V, Johnson and Johnson, and Sinopharm vaccinees. Graphs represent the correlation of SARS-CoV-2 specific spike-protein binding IgG and IgA with age in Moderna (**A**, **B**), BioNTech (**C**, **D**), AstraZeneca (**E**, **F**), Sputnik-V (**G**, **H**), Johnson and Johnson (**I**, **J**), and Sinopharm (**K**, **L**) vaccinees. Dots represent individual donors. Indicated r values were calculated using Spearman’s rank-order analysis.

To determine the impact of age on vaccine induced antibody production, Spearman’s correlation test was used to investigate the relationship between the age of the vaccinees and the amplitude of SARS-CoV-2 specific IgA and IgG expression after vaccination with Moderna, BioNTech, AstraZeneca, Johnson, and Johnson, Sputnik-V and Sinopharm vaccines. No correlation was seen between age and SARS-CoV-2 specific antibody expression in the Moderna, BioNTech, and AstraZeneca groups (**Figures 4A–F**). In sera of Sputnik-V vaccinees, a moderate negative antibody-age correlation was observed for IgA (ρ = -0.3917), whereas a trend was visible for IgG (ρ = -0.2540) (**Figures 4G, H**). In the Johnson and Johnson group, a moderate negative correlation was seen for IgG (ρ = -g0.3936) was seen between age and SARS-CoV-2- specific IgG expression (**Figures 4I, J**). A stronger negative correlation was seen between the expression SARS-CoV-2-specific IgG and age in the Sinopharm group (ρ = -0.6977) (**Figures 4K, L**).

### Low SARS-CoV-2 Specific Antibody Expression and Neutralizing Potential in Older Johnson and Johnson and Sinopharm Vaccinees

**Figure 5:**
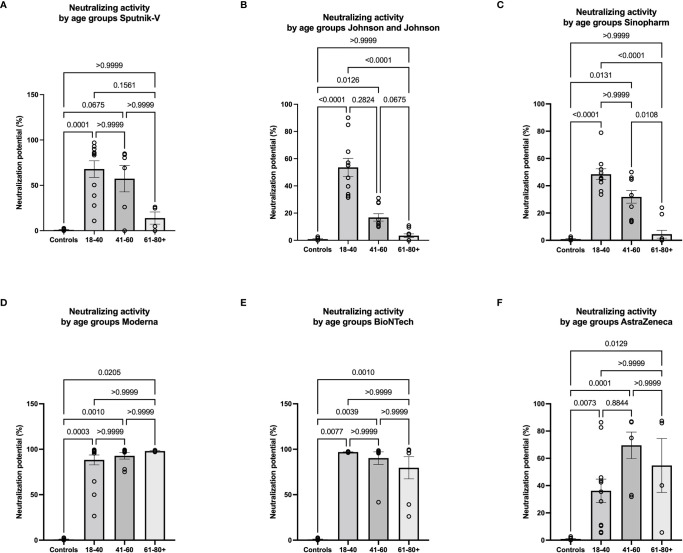
Low neutralizing antibody response in older vaccinees of Sputnik-V, Johnson and Johnson, and Sinopharm groups. Graphs represent the relative expression of neutralizing antibodies observed using sera from Sputnik-V **(A)**, Johnson and Johnson **(B)**, Sinopharm **(C)**, Moderna **(D)**, BioNTech **(E)**, and AstraZeneca **(F)** vaccinees in 3 different age categories: 18-40, 41-60, and 61-80+ years old. Each symbol represents individual donors. Indicated p values were calculated using Kruskal-Wallis ‘test followed by Dunn’s comparison *post hoc* to compare all groups. Bars represent the mean percentages of relative neutralizing antibody expression ± SEM. Significance is accepted if p <0.05.

The strongest negative correlations between age and antibody expression were observed in the Sputnik-V, Johnson and Johnson, and Sinopharm groups. To further investigate the impact of age on the humoral immune response in these groups, the relative expression of neutralizing antibodies was analyzed in 3 different age categories: 18-40, 41-60, and 61-80+. For Sputnik-V, Johnson and Johnson, and Sinopharm groups, no significant neutralization potential was observed in the elderly between 61and 80 years old. Low to moderate neutralization potentials were measured in the age categories of 41 to 60 years old. Differences to the control group were significant in the age category of 18-40 years old in all groups of vaccinees and in the category of 41-60 years old for Johnson and Johnson and Sinopharm vaccinees. In contrast, a trend was seen for Sputnik-V in this age category (**Figures 5A–C**). In contrast, neutralization potentials were statistically comparable in the different age categories in sera of Moderna, BioNTech, and AstraZeneca vaccinees (**Figures 5D–F**).

### Robust Antibody Production and Neutralization Potential After AstraZeneca-mRNA Vaccine Combinations

**Figure 6:**
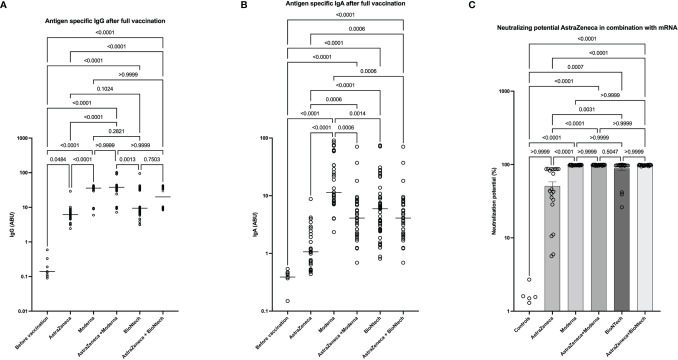
High neutralizing antibody expression in AstraZeneca- mRNA vaccine combinations. Graphs represent the expression of SARS-CoV-2 specific spike-protein binding IgG **(A)**, IgA **(B)**, and neutralizing antibody expression **(C)** observed in the sera of people that received homologous (AstraZeneca, Moderna, or BioNTech) ‘s COVID-19 vaccines or heterologous vaccine doses (AstraZeneca-Moderna or AstraZeneca-BioNTech). Each symbol represents individual donors. Indicated p values were calculated using Kruskal-Wallis ‘test followed by Dunn’s comparison *post hoc* to compare all groups. Bars represent the median **(A, B)** of SARS-CoV-2 spike protein-specific antibody expression in each group in arbitrary binding units (ABU) or the means ± SEM percentages of relative neutralizing antibody expression **(C)**. Significance is accepted if p <0.05.

We next compared the antibody responses with a double shot of AstraZeneca, Moderna, and BioNTech with the combinations AstraZeneca/Moderna or AstraZeneca/BioNTech. Our data showed that the combinations of AstraZeneca with a second dose of Moderna or BioNTech are significantly more effective at inducing SARS-CoV-2 specific IgG and IgA compared to 2 doses of AstraZeneca. In addition, IgA levels were higher in the homologous Moderna group compared to the AstraZeneca-Moderna group. No significant difference was seen between the AstraZeneca-BioNTech group and the homologous BioNTech group (**Figures 6A, B**). These results were also reflected by the neutralization data. Indeed, neutralization potentials in sera of AstraZeneca/Modern and AstraZeneca/BioNTech groups were significantly higher compared to the groups who received 2 doses of AstraZeneca. In addition, while few individuals of the BioNTech group presented neutralization potentials lower than 70% in the AstraZeneca and BioNTech groups, all tested volunteers exhibited in the AstraZeneca/BioNTech group neutralizing potentials above 95% (**Figure 6C**).

## Discussion

In this study based on 365 vaccinated participants, we showed that post-vaccine anti-spike IgG responses significantly vary according to the vaccine type. mRNA vaccines from Moderna and BioNTech induced the highest amount of spike-specific IgG and IgA antibodies and a high serum neutralization potential. High antibody levels in mRNA vaccinees were also observed in other studies (26). The data suggest a high seroconversion and antibody-mediated virus neutralization potential in mRNA-vaccinated individuals.

Comparatively, DNA vectored vaccines Sputnik-V and AstraZeneca induced similar amounts of SARS-CoV-2 specific IgG. Interestingly, higher IgA expression was seen in the Sputnik-V group compared to the AstraZeneca group. Both groups, however, presented similar levels of neutralizing antibodies. These data can be explained by the fact that Sputnik-V and the AstraZeneca vaccine are very similar in their conception and principle of action. For both vaccines, the gene coding for the SARS-CoV-2 spike protein is introduced into an adenovirus vector. The main difference between these two vector vaccines is that Sputnik-V uses two different vectors, the rAd26 for priming and Ad5 in the booster dose (27), while AstraZeneca includes the spike protein gene in the ChAdOx1 viral vector (28, 29).

Our results clearly showed a weaker performance for the Johnson and Johnson vaccine compared to mRNA and the other DNA vectored vaccines. Indeed, the Johnson and Johnson vaccine induced relatively low spike-specific IgG, and the sera of vaccinees from this group exhibit no significant neutralizing potential compared to the unvaccinated controls. Similar findings were obtained in a larger survey by Self et al., where postvaccination anti-spike and anti-RBD IgG levels were seen to be significantly lower in persons vaccinated with Johnson and Johnson than Moderna or Pfizer-BioNTech vaccines (26). This weak performance can be explained by the fact that the Johnson and Johnson vaccine required only one dose. In line with our data, the American Food and Drug Administration (FDA) has recently amended the Emergency Use Authorizations (EUA) for the Janssen COVID-19 vaccine to include the use of a booster dose to be administrated to all recipients of the Johnson and Johnson vaccine. They may choose to receive either an additional full dose of Janssen’s vaccine or a full dose of an mRNA vaccine (30).

An even weaker performance was observed for the Sinopharm vaccine. In line with our data, a recent study in Bahrain showed that in a group of 22 persons vaccinated with a double dose of the Sinopharm vaccine, 20 were infected with SARS-CoV-2 (31). Saeed et al., after analyzing the expression of spike-specific antibody levels in 2868 COVID-19 vaccinated individuals with the Sinopharm vaccine in Iran, came to the conclusion that two doses of Sinopharm may not be adequate to provide long-lasting immunity against SARS-CoV-2 (32).

It is, however, noticeable that the Sinopharm vaccine used whole inactivated SARS-CoV-2 viral particles (33) so that our analyses of spike protein reactivity may miss the full extent of immune reactions to this vaccine. Further investigations are required to explore the responses to other viral proteins. Interestingly, despite its weak performance in terms of neutralizing antibody induction, our data indicate that the Sinopharm vaccine has the mildest side effects compared to the other vaccines. These data align with recent findings suggesting that both doses of the Sinopharm COVID-19 vaccine induce mild and common side effects (34, 35).

Nonetheless, correlation analyses of antigen-specific antibody expression with the age of the vaccinees revealed negative correlations between age and antibody expression, especially in Sputnik-V, Johnson and Johnson, and Sinopharm groups. Further analyses revealed that younger vaccinees with 18-40 years in these groups exhibit significant antibody and neutralizing potential compared to older vaccinees. In the age group of 41–60-year-olds, the antibody expression and neutralizing activity were lower but still significant compared to the unvaccinated controls. Thus, while neutralization potential in 18-60 years old Johnson and Johnson and Sinopharm vaccinees was significant, no significance was seen when considering older adults (60-80+ years). Our results on the Sinopharm vaccine are in line with recent data by Ferenci et al., indicating that antibody production after BBIBP-CorV vaccination was strongly reduced with increasing age (35).

Our data also suggest a higher risk of post-vaccination COVID-19 infection in this age category after Sputnik-V, Johnson and Johnson, and Sinopharm vaccines. This finding, together with the emergence of new virus variants, is very worrying since this very same population of the elderly is considered at high risk of developing severe forms of COVID-19. The negative impact of immune senescence on vaccine efficacy is well known in both human and animal models (36, 37). In Influenza vaccination, for example, it was shown that age-dependent reduction of the expression of critical regulators of B cell maturation and class switch recombination such as Blimp-1, E47, and AID, leads to the production of fewer functional antibodies in the elderly (38). However, such an age-dependent reduction of B cell functions alone is insufficient to explain the weaker SARS-CoV-2 specific antibody induction in older DNA-vectored vaccinees. Indeed, in our investigation, mRNA vaccinees seem to be unaffected. Additional mechanisms inherent to the dose and type of vaccines may contribute to this weak performance in older DNA vectored vaccinees. One factor could be the adenovirus vectors themselves. Indeed, emerging data suggest that immune responses to proteins encoded by the adenovirus vectors reduce antibody responses to the spike protein (39). It may be assumed that older vaccinees who have probably experienced several adenovirus infections might be more likely to exhibit cross-reactive immune responses against adenovirus vectors. To minimize this risk, manufacturers have used different strategies. Johnson & Johnson and AstraZeneca vaccines employed adenoviral strains (ChAd26 and ChAdY25, respectively) exclusively found in chimpanzees (40, 41). In contrast, the Sputnik V vaccine was developed using two different human adenoviral vectors for the first (rAd26) and the second (rAd5) vaccine dose (42). Despite these preventive measures, anti-vector immunity may at least partially contribute to the weaker performance of adenovirus-vectored vaccines, particularly observed in older vaccinees. Further investigations are required to fully elucidate the underlying mechanisms of the observed lower antibody response in this group of vaccinees. Implications for the millions of people who received these vaccines worldwide also need to be addressed. Booster doses with the more effective mRNA vaccines should be considered.

During our survey, the German Standing Vaccination Committee (STIKO), taking into account concerns after several reports of rare but serious blood clots in young adults (43, 44), recommended after AstraZeneca a second dose of one of the 2 available mRNA vaccines to individuals that received the first dose of AstraZeneca. We therefore also analyzed the antibody responses after this mixed vaccination strategy. The data clearly suggest a more robust SARS-CoV-2 specific IgG and IgA expression after this vaccination schedule. Similar data were found in Spain, where preliminary data on 600 AstraZeneca primed vaccinees demonstrated that a BioNTech second dose remarkably boosted antibody responses (45). Our data further confirmed that the mix-and-match COVID-19 vaccination strategy triggered a stronger antibody production than two doses of a single vaccine. Noticeable was, however, that Moderna vaccinees conserved the highest SARS-CoV-2 specific IgA expression compared to all other groups.

The findings in this report are subject to three major limitations. First, antibody specificity and neutralization potential were not tested against emerging variants of concern. Indeed, an increasing concern is whether the vaccines currently available can protect against emerging SARS-CoV-2 variants (46–48). The study was largely performed before the predominance of the Delta (B.1.617.2) and the emergence of the Omicron variants in Europe (5). Further investigations are required to analyze in-depth the antibody responses to different SARS-CoV-2 variants. Second, the present study did not investigate the durability of neutralizing antibody expression after full vaccination with the different vaccines and vaccine combinations. Emerging lines of evidence suggest that antibody levels after COVID-19 vaccination may drop at different rates depending on various factors, including the type of vaccine, infection before or after vaccination, age, sex, T-cell response, and the interval between vaccine injections (49, 50). Therefore, additional investigations are required to better define the stability of immune effectors after COVID-19 vaccination. Third, while our data focus on antibody responses, T cell reactivity and T cell memory might represent another important mechanism for long-lasting vaccine-induced protection.

The present study compared the efficacy of the 6 major COVID-19 vaccines currently available (Moderna, BioNTech, AstraZeneca, Johnson and Johnson, Sputnik-V, and Sinopharm’s COVID-19 vaccines). Our findings suggest that mRNA vaccines induced the highest titers of SARS-CoV-2 specific neutralizing antibodies. While all 6 vaccines have moderate reactogenicity and induce functional neutralizing antibodies in vaccinees, low antibody-mediated protection is seen in the elderly vaccinated with DNA-vectored vaccines. Our data also demonstrated that heterologous vaccination strategies using priming with the AstraZeneca followed by a boost with an mRNA vaccine induced more robust antibody expression and virus neutralization potential compared to their homologous counterparts.

## Data Availability Statement

The original contributions presented in the study are included in the article/supplementary material. Further inquiries can be directed to the corresponding author.

## Ethics Statement

The studies involving human participants were reviewed and approved by Ethical board of the University Hospital Bonn. Ethical board of the Faculty of Medicine of the University of Novi Sad. The patients/participants provided their written informed consent to participate in this study.

## Author Contributions

TA and AH conceived the study and were in charge of overall coordination. TA, JM, LS, and MB carried out the experiments. JM, MP, CB, PK, and AT contributed to sample collection. TA, AH, JM and MP analyzed the data and contributed to the interpretation of the results. TA wrote the manuscript. All authors provided critical feedback and helped shape the research, analysis, and manuscript.

## Funding

AH is funded by the Deutsche Forschungsgemeinschaft (DFG, German Research Foundation) under Germany’s Excellence Strategy – EXC2151 – 390873048”. The funders had no role in study design, data collection, analysis, decision to publish, or manuscript preparation.

## Conflict of Interest

The authors declare that the research was conducted in the absence of any commercial or financial relationships that could be construed as a potential conflict of interest.

## Publisher’s Note

All claims expressed in this article are solely those of the authors and do not necessarily represent those of their affiliated organizations, or those of the publisher, the editors and the reviewers. Any product that may be evaluated in this article, or claim that may be made by its manufacturer, is not guaranteed or endorsed by the publisher.
